# Reporting Requirements, Confidentiality, and Legal Immunity for Physicians Who Report Medically Impaired Drivers

**DOI:** 10.1001/jamanetworkopen.2023.50495

**Published:** 2024-01-05

**Authors:** Elaine M. Tran, Jeffrey E. Lee

**Affiliations:** 1 Shiley Eye Institute, Department of Ophthalmology, University of California, San Diego, La Jolla

## Abstract

**Importance:**

Physicians play an important role in assessing patients’ ability to drive. There is a dearth of peer-reviewed information on policies regarding physician reporting of medically impaired drivers.

**Objective:**

To investigate state reporting requirements and the availability of confidentiality and legal immunity for physicians who report medically impaired drivers.

**Design, Setting, and Participants:**

This cross-sectional study was conducted from November 1 to 30, 2022, in 3 rounds. First, all 50 US states’ Department of Motor Vehicle (DMV) websites were systematically reviewed. Second, DMV staff from each state were surveyed via telephone. Third, each state’s legal codes for driver licensing were reviewed.

**Main Outcomes and Measures:**

Outcome measures included the percentage of states with mandatory and voluntary reporting policies, reporting instructions on DMV websites, anonymous reporting options, and legal immunity for reporting physicians, in addition to characteristics of states’ mandatory reporting policies (ie, types of medical conditions that require reporting) and policies surrounding the confidentiality of reports. The data were analyzed using descriptive statistics.

**Results:**

One-third of state DMV websites (17 [34%]) lacked instructions regarding physician reporting. Six states had mandatory reporting requirements; 4 of these states only required reporting of conditions characterized by lapses of consciousness. Only 3 states (6%) accepted anonymous reports, and 7 states (14%) deemed physician reports of medically impaired drivers confidential without exception. Nearly one-third of states (15 [30%]) deemed reports by physicians confidential, with the exception that reported drivers could find out who reported them if they asked for a copy of the reporting form. Most states (37 [74%]) had statutes that protected physicians from liability related to reporting medically impaired drivers.

**Conclusions and Relevance:**

This cross-sectional study of state reporting requirements regarding medically impaired drivers found many differences in state policies regarding mandatory reporting and the conditions that require reporting. There was also limited availability of online reporting instructions, anonymous reporting options, and legal protections for reporting physicians.

## Key Points

**Question:**

What are the state reporting requirements for physicians who report medically impaired drivers, and are confidentiality and legal immunity available to them?

**Findings:**

In this cross-sectional study of all 50 US states’ Motor Vehicle Departments, only 6 US states required mandatory reporting, 3 states accepted anonymous reports, 7 states deemed physician reports confidential without exception, and 37 states granted legal immunity to reporting physicians. One-third of state Department of Motor Vehicle websites lacked instructions regarding physician reporting of medically impaired drivers.

**Meaning:**

This study found variations in state reporting requirements for physicians who treat medically impaired drivers and identified several potential barriers to physician reporting.

## Teaser

This cross-sectional study surveyed the Department of Motor vehicle websites in all 50 US states to investigate state reporting requirements and the availability of confidentiality and legal immunity for physicians who report medically impaired drivers.

## Introduction

Physicians play an important role in assessing patients’ ability to drive.^1,2^ Across a variety of medical specialties, including ophthalmology, emergency medicine, neurology, and primary care, physicians may encounter patients who drive despite having medical conditions that may make it unsafe to do so. Physicians have an opportunity and, in some states, an obligation to report medically impaired drivers to licensing agencies to reduce injuries and deaths from preventable motor vehicle crashes.

Physicians face a variety of logistical and ethical barriers when it comes to reporting medically impaired drivers. Physicians may fear that reporting will damage the patient-physician relationship. They may also lack training on what types of conditions to report and whether reporting in their state is mandatory or voluntary. In addition, physicians may feel uneasy about the confidentiality of the reporting process and the potential legal ramifications if disgruntled patients pursue legal action against them. The present study aims to address these barriers and concerns. To our knowledge, the most recent peer-reviewed study on these topics was published in 2011,^3^ which may predate many updates to state legal codes and Department of Motor Vehicle (DMV) websites.^4^ We synthesized data from all 50 US states’ DMV websites, telephone calls to DMV representatives, and state laws to present a review of mandatory and voluntary physician reporting requirements, options to report anonymously, the confidentiality of physician reports, and the availability of legal immunity for physicians who report medically impaired drivers.

## Methods

We identified 3 sources of information to examine the current requirements for reporting medically impaired drivers. First, to identify the state DMV websites that describe physician reporting of medically impaired drivers, Google searches were conducted with the following search terms: *state name*, *Department of Motor Vehicles*, *physician*, *reporting*, *medically*, *impaired*, and *driver*. Second, each DMV’s telephone number was collected from these websites. Automated DMV voice messaging systems were navigated to reach DMV licensing telephone representatives who helped direct the call to the office that manages physician reporting of medically impaired drivers. Third, to identify the section of each state’s legal code that describes physician reporting of medically impaired drivers, Google searches were conducted with the following search terms: *vehicle*, *legal*, *law*, *code*, *department of motor vehicles*, *physician*, *reporting*, *medically*, *impaired*, *driver*, and the state name. The University of California San Diego institutional review board determined that this study did not require institutional review board approval as the study did not meet the definition of human participants research. This study followed the Strengthening the Reporting of Observational Studies in Epidemiology (STROBE) reporting guideline for cross-sectional studies.^5^

From November 1 to 30, 2022, information was collected from these sources regarding whether reporting was mandatory or voluntary, options to report anonymously, medical conditions that need to be reported, how to report, the confidentiality of reports, exceptions to confidentiality, and legal immunity for reporting physicians.

### List of Questions for DMV Staff

Is physician reporting of medically impaired drivers mandatory or voluntary?
If it is mandatory, who needs to be reported, how long do physicians have to report, and what is the penalty for not reporting?
What is the process of reporting a medically impaired driver? Are there any forms to fill out? Where should the forms or written letters be sent?
Is there an option to report drivers anonymously?
Is the name of the reporting physician kept confidential?
If the reported driver asks the DMV who reported them, will DMV staff members reveal the name of the reporting physician?
If the reported driver has their drivers’ license revoked and they appeal the DMV decision and the case goes to court, will the reporting physician’s identity be revealed?
Are there any legal protections or legal immunity for physicians who report medically impaired drivers?
Who should be contacted to answer more questions about the reporting process?

### Statistical Analysis

The data were analyzed using descriptive statistics. Our statistical analysis was performed on December 3 and 10, 2022, using Microsoft Excel, version 16 (Microsoft Corp).

## Results

### Mandatory Reporting

Six states had mandatory reporting requirements (Table 1). Physicians in California, Delaware, Nevada, and New Jersey were required to report conditions that were characterized by lapses of consciousness, including epilepsy. In comparison, physicians in Oregon and Pennsylvania were required to report a much wider range of impairments, including poor visual acuity and visual field loss, memory loss in Alzheimer disease, and contemplation of suicide. Penalties for failure to report included $50 for each violation in New Jersey and possible conviction of a summary criminal offense in Pennsylvania. In addition to requirements to report medically impaired drivers via forms or written letters, physicians in Nevada had additional duties to educate patients about the dangers of operating a motor vehicle with their medical condition.

**Table 1.** Details on Mandatory Reporting Requirements in 6 US States

State	Reporting information on website	Mandated reporters	Mandatory conditions to report	Ages of reportable patients, y	Reporting time frame	Penalty for not reporting
California	Yes	Physicians	Conditions characterized by lapses of consciousness	≥14	14 d	Unspecified
Delaware	No	Physicians	Losses of consciousness due to a central nervous system disease	All ages	1 wk	Fine of $5-$50 and costs for failure to report
Nevada	No	Physicians, advanced practice registered nurses	Epilepsy	≥14	Unspecified	Unspecified
New Jersey	Yes	Physicians	Recurrent convulsive seizures or recurrent periods of unconsciousness or impairment or loss of motor coordination due to conditions, such as epilepsy	≥16	24 h	Fine of $50
Oregon	Yes	Clinicians in primary care, emergency medicine, vision care; those of patients without primary care clinicians	“Severe and uncontrollable” functional and/or cognitive impairments resulting from a medical condition (ie, visual acuity and field of vision, strength, motor planning and coordination, peripheral sensation, attention, judgment, reaction time, memory, and loss of consciousness)	≥14	Unspecified	Unspecified
Pennsylvania	Yes	All health care personnel	Disorders characterized by lapses of consciousness or other mental or physical disabilities (ie, visual acuity and field of vision, seizure disorder, unstable diabetes, cardiovascular conditions, cerebral vascular insufficiency, drugs, hallucination, suicidality)	≥15	10 d	Physicians could be convicted of a summary criminal offense

### Anonymity and Confidentiality

Among all 50 US states, only 3 (6%) accepted anonymous reports (Table 2). Furthermore, only 7 states (14%) deemed physician reports of medically impaired drivers confidential without exception. Nearly one-third of states (15 [30%]) deemed physician reports confidential, with the exception that drivers could learn the name of the reporting physician, request copies of the reporting form, or request their full driving record (which includes a copy of the reporting form). Most states (37 [74%]) deemed reports by physicians confidential, with the exception that reporting physicians’ identities may be revealed in judicial proceedings. We identified discrepancies in website and DMV staff statements on confidentiality practices. For instance, in Wyoming, while the reporting form stated that the report is confidential, DMV staff said that they would tell the driver the name of the reporting physician if requested and if the case goes to court.

**Table 2.** Reporting in 50 US States—Mandatory vs Voluntary, Confidentiality, Legal Immunity

State	Mandatory reporting	Accepts anonymous reports	Confidential without exception	Confidential except driver could receive report if requested	Confidential except in court	Not confidential	Immune from liability	Not immune from liability	Not liable if do not report
Alabama	N	N	N	Y	Y	N	Y	N	N
Alaska	N	N	N	N	Y	N	N	Y	N
Arizona	N	N	N	Y	Y	N	Y	N	N
Arkansas	N	N	N	Y	N	N	Y	N	N
California	Y	N	N	N	Y	N	Y^a^	N	N
Colorado	N	N	N	Y	Y	N	Y	N	N
Connecticut	N	N	N	Y	Y	N	Y	N	N
Delaware	Y	N	N	N	Y	N	Y^a^	N	Y
Florida	N	N	Y	N	N	N	Y	N	N
Georgia	N	N	N	N	Y	N	Y	N	N
Hawaii	N	N	N	N	Y	N	N	Y	N
Idaho	N	N	N	N	N	Y	Y	N	N
Illinois	N	N	N	N	Y	N	Y	N	N
Indiana	N	N	N	N	Y	N	Y	N	N
Iowa	N	N	Y	N	N	N	Y	N	N
Kansas	N	N	N	N	Y	N	Y	N	N
Kentucky	N	N	N	N	N	Y	Y^b^	N	N
Louisiana	N	N	N	N	Y	N	Y	N	N
Maine	N	N	N	Y	Y	N	Y	N	N
Maryland	N	Y	N	N	Y	N	Y^c^	N	N
Massachusetts	N	N	N	Y	Y	N	Y	N	Y
Michigan	N	N	Y	N	N	N	Y	N	Y
Minnesota	N	N	N	Y	Y	N	Y	N	Y
Mississippi	N	N	N	N	Y	N	N	Y	N
Missouri	N	N	N	N	Y	N	Y	N	N
Montana	N	N	N	Y	Y	N	Y	N	N
Nebraska	N	N	N	N	Y	N	N	Y	N
Nevada	Y	N	N	Y	Y	N	N	Y	Y
New Hampshire	N	N	N	N	N	Y	Y	N	N
New Jersey	Y	N	N	N	N	Y	Y^a^	N	N
New Mexico	N	Y	Y	N	N	N	Y	N	N
New York	N	N	N	N	N	Y	N	Y	N
North Carolina	N	N	Y	N	N	N	Y	N	Y
North Dakota	N	N	N	Y	N	N	Y	N	N
Ohio	N	N	Y	N	N	N	N	Y	N
Oklahoma	N	N	N	N	N	Y	Y	N	N
Oregon	Y	N	N	N	Y	N	Y^a^	N	Y
Pennsylvania	Y	N	N	N	Y	N	Y^a^	N	N
Rhode Island	N	N	N	Y	Y	N	Y	N	Y
South Carolina	N	N	N	N	Y	N	N	Y	N
South Dakota	N	N	N	N	Y	N	N	Y	N
Tennessee	N	N	N	Y	Y	N	N	Y	N
Texas	N	Y	N	N	Y	N	Y	N	N
Utah	N	N	N	N	Y	N	Y	N	N
Vermont	N	N	N	N	Y	N	N	Y	N
Virginia	N	N	Y	N	N	N	Y	N	N
Washington	N	N	N	N	N	Y	N	Y	N
West Virginia	N	N	N	Y	Y	N	Y	N	N
Wisconsin^d^	N	N	N	N	Y	N	Y	N	N
Wyoming	N	N	N	Y	Y	N	N	Y	N
No. (%) (N = 50)	6 (12)	3 (6)	7 (14)	15 (30)	34 (68)	7 (14)	37 (74)	13 (26)	8 (16)

Abbreviations: N, no; Y, yes.

^a^ These states required mandatory reporting and their legal codes only specified legal immunity for reporting conditions that were required to be reported; they did not specify legal immunity for reporting conditions that could be voluntarily reported.

^b^ Kentucky state law only specifies legal immunity for reporting seizures.

^c^ Maryland state law only specifies legal immunity for reporting disorders characterized by lapses of consciousness and corrected visual acuity that does not meet vision requirements.

^d^ Wisconsin MV3454 Pledge of Confidentiality can be filled out to make a report confidential such that Department of Motor Vehicle staff cannot release the reporting form to the driver.

### Legal Immunity

Of all 50 US states, 37 (74%) had state statutes that protected physicians from liability related to reporting medically impaired drivers (Table 2). California, Delaware, New Jersey, Oregon, and Pennsylvania provided legal immunity for reporting conditions that fall under mandatory reporting, but their legal codes did not specify legal immunity for reporting other conditions. Only 8 states’ legal codes (16%) included clauses that protect physicians from liability if they do not report medically impaired drivers.

### Instructions on Reporting

The eTable in Supplement 1 provides detailed instructions on how to report a medically impaired driver for physicians in each US state, including links to reporting forms; email, fax, or mailing directions; and telephone numbers to Medical Advisory Board or Medical Unit representatives, if available. Approximately one-third of state DMV websites (17 [34%]) did not provide any information or instructions regarding physician reporting; for these states, the instructions included in the eTable in Supplement 1 were deduced from telephone calls to their respective DMVs.

## Discussion

In this study, we identified many differences in state policies regarding physician reporting of medically impaired drivers. We also identified the limited availability of online reporting instructions, anonymous reporting options, and legal protections for reporting physicians.

Geographically, the 6 mandatory reporting states were clustered in the Pacific West and Northeast regions. Although reporting was mandated in only 6 states (12%), mandatory reporting policies may affect a disproportionate fraction of the US population; according to the US Census Bureau, 21% of US residents lived in these states in 2022.^6^ We noted considerable variation in the definition of a medically impaired driver. Of the states that required mandatory reporting, two-thirds only required reporting of conditions characterized by lapses of consciousness. Although Nevada specifically requires epilepsy to be reported, other states named a variety of other conditions involving lapses of consciousness, including hypoglycemia associated with diabetes and narcolepsy. Other states seemed to view conditions, such as vision impairment and dementia, on a continuum and encouraged monitoring for features of these conditions, such as advanced visual field loss.^7^ Furthermore, many states emphasized that age alone is not a valid reason to report a driver.

Ophthalmologists, among other specialists, are routinely asked to evaluate patients’ fitness to drive; however, education on policies and best practices surrounding medically impaired driver reporting is not a standard part of graduate medical education. Furthermore, studies have reported that clinicians in family medicine, geriatrics, ophthalmology, and optometry are unaware of state reporting requirements and actions to take if a patient appears unsafe to drive.^4,8,9,10,11,12^ This lack of awareness may be explained in part by our finding that one-third of DMV websites have no information regarding physician reporting of medically impaired drivers. In addition, there is a dearth of published information on this topic. The National Highway Traffic Safety Administration may be well positioned to help state DMVs establish best practices on how to present instructions and resources regarding physician reporting of medically impaired drivers; furthermore, state legislatures hold the power to regulate these policies and provide resources to make reporting systems available. One risk associated with reporting a medically impaired driver is potential damage to the physician-patient relationship.^9^ Physicians may fear that reporting a medically impaired driver may lead patients to seek care from other clinicians, withhold information regarding their symptoms, and, potentially, retaliate against them. As such, physicians may be more inclined to report at-risk drivers if there are avenues to report drivers anonymously or there are protections in place to prevent reported drivers from learning the identity of the reporting physician. Our study showed that only 3 states permitted anonymous reporting. Several states justified the lack of anonymous reporting options by citing the importance of verifying the reporting physicians’ credentials, which can help shed light on the veracity of reports and curtail reports submitted with malicious intent. State DMVs may consider developing procedures to verify reporting physicians’ credentials, while omitting the identity of reporting physicians in communications with reported drivers.

Although many states deemed the reporting process confidential, many states had avenues for drivers to learn the identity of reporting physicians, which we call *exceptions to confidentiality*. We also identified discrepancies regarding confidentiality policies and practices when comparing DMV website information with the verbal reports of DMV staff in Wyoming. As such, it is important for clinicians to cross-reference sources when researching their state’s reporting procedures. Similarly, we encourage state DMVs to ensure that website information, state statutes, and the practices of DMV staff are transparent, accurate, and consistent.

The American Medical Association and the American Academy of Neurology, among other professional organizations, recognize the importance of safeguarding physicians from liability when they report patients in good faith.^1,13^ A study of vision care clinicians reported that they cite liability risk for reporting and for not reporting as barriers to reporting unsafe drivers.^9^ At the time of this study, universal legal immunity did not exist in the US. Legal immunity for not reporting may be just as important as immunity for reporting. Our study suggests that, in 42 states, physicians who decide not to report medically impaired drivers may be vulnerable to lawsuits by third parties who are injured by the impaired driver. The legal environment surrounding physician reporting continues to evolve, and physicians may benefit from reviewing summaries of legal cases involving physicians who have reported or not reported medically impaired drivers.^14^

The conclusions of past research on the effectiveness of physician reporting have been mixed. Some studies have found that mandatory physician reporting laws do not have a significant association with hospitalizations due to motor vehicle crashes,^8^ while others report that physician reporting is associated with reduced road crashes and traffic violations.^15,16,17,18^ Another often-cited concern with physician reporting is that it could result in inherently safe drivers losing their driving privileges.^15,19^ Physicians may consider the similarities of mandated reporting of communicable diseases with the reporting of medically impaired drivers. Both appeal to physicians’ duties to protect public health and safety. The American Medical Association offers ethical guidelines to help guide physicians in making these difficult decisions.^1,2^ Physicians are encouraged to educate patients and their families about the risks of driving and state laws regarding licensing requirements.^1,20^

Nonetheless, physicians should be reassured that physicians in the US are not directly responsible for issuing or revoking driving privileges. Rather, in many states, physician reports prompt their state DMV to conduct a driving reexamination that may then compel the DMV to reinstate, set limitations to, or suspend a patient’s driving privileges.^2^ In many states, the final decision is made by a medical advisory board that is staffed by physicians. A study of older drivers’ opinions about driving cessation reported that most older drivers believe that physicians, rather than state DMVs or police officers, should determine license revocation.^21^ Such findings reinforce the important role that physicians can play in identifying medically impaired drivers and counseling their patients about safe driving.^21,22^

### Limitations

This study has several limitations. First, this is a cross-sectional study; we collected data in November 2022 and recognize that state laws and policies may change over time. Compared with the results of a cross-sectional study on this topic performed in 2010, our 2022 study found that the same 6 states had mandatory reporting requirements and that 37 states provided immunity for reporting physicians compared with 32 in 2010.^3^ Therefore, clinicians and state medical societies should try their best to stay up to date with local regulations and contact DMVs directly if they have questions or concerns. Second, we surveyed 1 DMV staff member from each state; while it is possible that surveying additional DMV staff members may have yielded different results, many DMVs only had 1 point person for physician reporting of medically impaired drivers. Third, for a small handful of states, legal codes were not available on government websites. However, for all those states, we were able to identify relevant legal codes on 2 websites^23,24^ that have been deemed reliable sources of federal and state statutes by the legal community.

## Conclusions

This cross-sectional study found a wide variation in state policies related to physician reporting of medically impaired drivers and found that many state DMV websites lacked information on these policies. In addition, physician reporting was neither universally confidential nor legally protected in the US. We encourage physicians to review the eTable in Supplement 1 as a starting point to understanding their state’s reporting procedures. We also encourage state DMVs to improve the delivery and reliability of website and telephone-based information on issues important to physician reporting, including confidentiality and legal immunity.

## Notes

Supplement 1. **eTable.** Instructions for Reporting in 50 US States

Supplement 2.
Data Sharing Statement
