# Supplementary material for: Reporting Requirements, Confidentiality, and Legal Immunity for Physicians Who Report Medically Impaired Drivers
Source: JAMA Netw Open. 2024 Jan 5;7(1):e2350495. doi: 10.1001/jamanetworkopen.2023.50495 (PMC10770772; doi:10.1001/jamanetworkopen.2023.50495)
Supplement: Supplement 1. — eTable. Instructions for Reporting in 50 US States [file jamanetwopen-e2350495-s001.pdf]

Supplementary Online Content

Tran EM, Lee JE. Reporting requirements, confidentiality, and legal immunity for physicians who report medically impaired drivers. *JAMA Netw Open.* 2024;7(1):e2350495. doi:10.1001/jamanetworkopen.2023.50495

eTable. Instructions for Reporting in 50 US States

This supplementary material has been provided by the authors to give readers additional information about their work.

© 2024 Tran EM et al. *JAMA Network Open.*

eTable. Instructions for Reporting in 50 US States

State Name	DMV Website Lacked Reporting Instructions	Instructions on How to Report a Medically Impaired Driver
**Alabama**	X	Send a letter on letterhead to the DMV Medical Unit via fax (334) 353-2008 or mail to PO box 1471 Montgomery, Alabama 36102.
**Alaska**		Send a completed Recommendation for Re-Examination Form (Form 411) (https://doa.alaska.gov/dmv/forms/pdfs/411%20-%20ReExam.pdf) to Anchorage Driver Services via email to doa.dmv.ads@alaska.gov, fax (907) 269-3774, or mail to Anchorage Driver Services 3901 Old Seward Highway, Suite 101 Anchorage, AK 99503. A letter can also be sent to Anchorage Driver Services. For questions, contact (907) 269-3770.
**Arizona**		Send a completed Physician Examination Report #96-0668 (https://apps.azdot.gov/files/mvd/mvd-forms-lib/96-0668.pdf) to the Medical Review Program via email to MedicalReview@azdot.gov or fax (602) 239-6288.
**Arkansas**		Send a completed Medical Evaluation Request form (https://www.dfa.arkansas.gov/images/uploads/driverServicesOffice/Initial_Evaluation-Family_Member_Request_3-30-2020.pdf) to the Office of Driver Services via email to arhearingofficers@dfa.arkansas.gov, fax (501) 683-0955, or mail to Driver Control P.O. Box 1272 Room 1070 Little Rock, AR 72203.
**California**		Mandatory Reporting: To report lapses of consciousness, send a completed Confidential Morbidity Report (Form 110C). Detailed instructions for the three most populous counties in CA: Los Angeles County send (http://publichealth.lacounty.gov/cdcp/docs/form_Physician_Report_to_LACoLHO&DMV_Lapses_of_Consciousness_Disorder_082718-fillable,Sig,mod100418.pdf) to the Los Angeles County Department of Public Health via fax 888-397-3778 or 213-482-5508; for questions, call 888-397-3993. Orange County send (http://www.ocagingservicescollaborative.org/wp-content/uploads/2014/08/Report-Form-for-DMV.pdf) to Epidemiology & Assessment (reports will be forwarded to the Department of Motor Vehicles) via fax (714) 564-4050; for questions, call (714) 834-8180. San Diego County send (https://www.sandiegocounty.gov/content/dam/sdc/hhsa/programs/phs/documents/CMRc.pdf) to County of San Diego Epidemiology via fax (858) 715-6458; for questions, call (619) 692-8499. Statewide Voluntary Reporting: To report other concerns regarding a patient's medical conditions that can impair driving, bring a completed Request for Driver Reexamination Form (DS 699) (https://www.dmv.ca.gov/portal/file/request-for-driver-reexamination-ds-699-pdf/) or written letter to the local Department of Motor Vehicles (DMV) office or mail to the local DMV, Driver Safety Office (addresses found on the DS 699 form).
**Colorado**	X	Send a request for re-examination due to physical or mental constraints via email to the Colorado DMV at dor_mvhelpdesk@state.co.us. The email should include the driver's name and date of birth, the reason for concern, and the name and relationship to the driver. For questions, email dor_mvhelpdesk@state.co.us.
**Connecticut**	X	Send a completed Impaired Driver Report (https://portal.ct.gov/-/media/DMV/20/29/P142ERpdf.pdf) or a letter written on physician letterhead via mail to DMV, Driver Services Division, 60 State Street, Wethersfield, CT 06161-1013.
**Delaware**		Mandatory Reporting: To report LOC/seizure activity, send a Medical Report of Physician's Findings form (MV 346) (https://www.dmv.de.gov/forms/driver_serv_forms/pdfs/dr_frm_mv346.pdf?cache=1669770487340) (driver signature not required) via mail to Medical Records Section -Driver Improvement Unit - PO Box 698 Dover, DE 19903-0698 or fax (302) 739-5667 with Attn: Medical Records Section. Voluntary Reporting: Reports

© 2024 Tran EM et al. *JAMA Network Open.*

		for all other conditions that can impair driving can be made via the same forms or the Report of Visual Status form (https://www.dmv.de.gov/forms/driver_serv_forms/pdfs/dr_frm_vision.pdf?cache=1669770490302?cache=1669789595366) which can be emailed to dmvmedicalsection@delaware.gov. For questions, call the Medical Section at (302) 744-2507 or email dmvmedicalsection@delaware.gov.
**Florida**		Send a completed Medical Reporting Form (#72190) (https://www.flhsmv.gov/pdf/forms/72190.pdf) or write a letter on letterhead to the Department of Highway Safety and Motor Vehicles via fax (850) 617-3944 or mail to Bureau of Motorist Compliance Medical Review Program Neil Kirkman Building, MS 86 Tallahassee, Florida 32399-0500. For questions, call (850) 617-3814.
**Georgia**		Send a completed Request for Driver Review Form (DDS 270) (https://dds.georgia.gov/medical-review-process) or letter via fax to (770) 344-3629 or mail Georgia Department of Driver Services c/o Medical Review Unit P.O. Box 80447 Conyers, GA 30013.
**Hawaii**	X	Send a letter to the Medical Advisory Board (MAB) including the patient/driver's full name, date of birth, if possible, their drivers license number and reason for concern via mail to Driver Licensing Section ATTN: Nilda Ocreto P.O. Box 30340 Honolulu, HI 96820-0340. The driver will later be required to have their doctor fill out Medical Report form HDOT-H2058 (https://hidot.hawaii.gov/highways/files/2018/05/MVSO-MAB-Medical-Report-DOT-H2058-2018-05-15-fillable.pdf). For questions, call the Medical Advisory Board at (808) 692-7656 or (808) 692-7655.
**Idaho**	X	Send an email including the driver's full name, driver's license number of social security number, date of birth, address, reason for evaluation, type of evaluation recommended, reporter name and signature to itdmeddesk@itd.idaho.gov. State statue (https://legislature.idaho.gov/statutesrules/idstat/title49/t49ch3/sect49-326/) states that before submitting a report, a physician should notify the patient or the patient's family of the physician's concerns about the patient's ability to drive. If the physician submits a report, the physician shall provide a copy of the report to the patient or to a member of the patient's family. For questions, call (208) 334-4443
**Illinois**	X	Send a completed Medical Report For Conditions That May Impair Driving Safely Form (https://www.ilsos.gov/publications/pdf_publications/dsd_dc163.pdf) to the Secretary of State Office via fax 217-785-3016 or mail to Secretary of State Medical Review Unit 2701 S. Dirksen Pkwy. Springfield, IL 62723. For questions, call Secretary of State Office's Medical Review Unit at (217) 782-7246.
**Indiana**		Send a completed Request for Driver Ability Review (Form 54750) (https://www.in.gov/bmv/licenses-permits-ids/driver-ability-program/) to the BMV via fax to (317) 974-1614 or mail to Indiana Bureau of Motor Vehicles Attn: Driver Ability Department 100 N Senate Ave RM 481 Indianapolis, IN 46204. For questions, call the Medical/Vision Unit at (785) 368-8971.
**Iowa**		Send a completed Request for Re-examination Form (Form 431030) (https://iowadot.seamlessdocs.com/f/RequestforReexamination) to the Iowa Department of Transpotation via fax (515) 239-1837 or mail Driver & Identification Services PO Box 9204 Des Moines, IA 50306-9204. For questions, call 515-244-8725 or email Driver.Services@iowadot.us.
**Kansas**		Send a completed Letter of Concern/Driver Evaluation Request Form (DC-10LOC) (https://www.ksrevenue.gov/pdf/DriverEvalRequest.pdf) to the Medical/Vision Unit via email KDOR_Medical.VisionUnit@ks.gov, fax (785) 296-5857, or mail Division of Vehicles Medical/Vision Unit PO BOX 2188 Topeka, KS 66601-2188.
**Kentucky**		Send a completed Medical Review Affidavit (TC 94-182) (https://transportation.ky.gov/Organizational-Resources/Forms/TC%2094-182.pdf) to via email to KYTC.MedicalReviewBoard@ky.gov, fax (844) 503-4111, or mail

© 2024 Tran EM et al. *JAMA Network Open.*

		Kentucky Transportation Cabinet, Department of Vehicle Regulation, Medical Review Board Office 200 Mero Street, Frankfort, KY 40622. For questions, call (502) 564-1257.
**Louisiana**		Send a completed Driver Behavior Report (DPSMV 3005) (https://public.powerdms.com/LADPSC/documents/351659) and mail to Attn: Driver's License Suspension Unit. Louisiana Department of Public Safety and Corrections. Office of Motor Vehicles PO Box 64886 Baton Rouge, LA 70896-4886.
**Maine**		Send a completed Driver Medical Evaluation form (CR-24) (https://www.maine.gov/sos/bmv/forms/CR24.pdf) or Eye Examination Form (MVE-103) (https://www.maine.gov/sos/bmv/forms/eyeform.pdf) for vision concerns or a written letters to the BMV via email medical.bmv@maine.gov, fax (207) 624-9319, or mail reply to: Bureau of Motor Vehicles, Medical Section 29 State House Station Augusta, Maine 04333-0029. For questions, call (207) 624-9000 ext. 52124.
**Maryland**		Send a completed Voluntary Physician/Healthcare Professional Referral Form (DC-220) (https://mva.maryland.gov/Documents/DC-220.pdf) or provide a statement on letterhead to the Maryland MVA via email: dwsmed@mdot.state.md.us, fax: 410-582-4936, or mail Maryland Motor Vehicle Administration Driver Wellness and Safety Division Attention: Nurse Case Review Manager 6601 Ritchie Highway, NE, Room 124 Glen Burnie, MD 21062. For questions, call (410) 768-7513.
**Massachusetts**		Send a completed Request for Medical Evaluation form (https://www.mass.gov/doc/request-for-medical-evaluation/download) to Registry of Motor Vehicles via mail to Attention: Medical Affairs P.O. Box 55889 Boston, MA 02205 or fax to (857) 368-0018. For questions, call at (857) 368-8020.
**Michigan**	X	Send a completed Request for Driver Evaluation Form (OC88) (https://www.michigan.gov/sos/-/media/Project/Websites/sos/27lawensn/OC88.PDF) via email to MedicalForms@Michigan.gov, fax to 517-335-2189, or mail to Michigan Department of State Driver Assessment Section P.O. Box 30810 Lansing, Michigan 48909-9832. For questions, call (517) 335-7051.
**Minnesota**	X	Send a completed Request for Examination of Driver Form (PS31924) (https://dps.mn.gov/divisions/dvs/forms-documents/Documents/DL-Request-Examination-of-Driver.pdf) and mail to Driver and Vehicle Services, Attn: Medical Unit, 445 Minnesota Street Suite 170, St. Paul, MN 55101-5170.
**Mississippi**		Send a notarized and signed Safety Concerned Letter via express mail to Driver Records Medical Division P.O. Box 1459 Canton, MS 39046. For questions, call (601) 487-7055.
**Missouri**		Send a completed Physician's Statement (Form 1528) (https://dor.mo.gov/forms/1528.pdf) via mail to Missouri Department of Revenue 301 West High Street - Room 470 Jefferson City, MO 65105-0200. For questions, call (573) 751-2730 or email dlbmail@dor.mo.gov.
**Montana**	X	Send a completed Driver Medical Evaluation form (20-1900) (https://dojmt.gov/wp-content/uploads/Driver-Medical-Evaluation.pdf) and email DriverLicense@mt.gov, fax to (406) 444-1631, or mail to Motor Vehicle Division Attn. Medical Unit P.O. Box 201430 Helena, MT 59620-1430. For questions, call (406) 444-3933.
**Nebraska**	X	Send a completed Physician Re-Examination Form (https://dmv.nebraska.gov/sites/dmv.nebraska.gov/files/doc/dls/forms/CitizenReexaminationReport.pdf) or write letter on letterhead and mail to DMV Drive Licsensing Division PO Box 94726 Lincoln, NE 68509-4726.
**Nevada**	X	For both mandatory and voluntary reporting, send a completed Confidential Physician’s Report (DLD-7) (https://dmv.nv.gov/pdfforms/dld7.pdf) https://dmv.nv.gov/pdfforms/dld23a.pdf or Eye Examination Certificate (DP18) (https://dmv.nv.gov/pdfforms/dp18.pdf) via fax to (775) 684-4829. For questions,

© 2024 Tran EM et al. *JAMA Network Open.*

		call driver license assessment department at (775) 684-4364 8 am (select option 2 twice) or email dlreviewweb@dmv.nv.gov.
**New Hampshire**	X	Send a written letter to NH Department of Safety Division of Motor Vehicles via mail to 23 Hazen Drive Concord, NH 03305. For questions, call (603) 227-4020.
**New Jersey**		Mandatory Reporting: To report patients who suffered recurrent losses of consciousness, send a completed Medical Emergency Report Form (MR-4) (https://www.state.nj.us/mvc/pdf/license/MVC-Form_MR-4.pdf) or a written letter via email to MVCMedicalReview@mvc.nj.gov or mail to Medical Fitness Review Unit P.O. Box 173 Trenton, New Jersey 08666-0173<br>Voluntary Reporting: Use the same form or submit a letter for voluntary reports of non-epilepsy conditions that may make a patient medically impaired to drive.
**New Mexico**	X	Send in a written letter to Htet Gonzales, the Director of the Motor Vehicle Department via mail to Attn: Drivers Services Bureau, Motor Vehicle Division, P.O. Box 1028, Santa Fe, NM 87504-1028.
**New York**		Send a completed Physician's Reporting Form (DS-6) (https://dmv.ny.gov/forms/ds6.pdf) via mail to Medical Review Unit, NYS Department of Motor Vehicles, 6 Empire State Plaza, Room 337, Albany, NY 12228. For questions, call the Medical Review Unit at (518) 474-0774, option #3.
**North Carolina**		Send a completed Request for Medical Evaluation form (https://www.ncdot.gov/dmv/downloads/Documents/medical-request.pdf) via fax to (919) 733-9569 or mail to DMV Medical Review Program, 3112 Mail Service Center, Raleigh, NC 27697.
**North Dakota**		Send a completed Medical Examination Report (SFN 4569) (https://www.dot.nd.gov/forms/sfn04569.pdf) viacor fax to (701) 328-0308 or mail to Driver License DIvison Attention: Medical Program Coordinator 608 East Boulevard Avenue Bismarck, ND 58505-0750. For questions, call (701) 328-4355.
**Ohio**	X	Send a completed Request for Statement of Physician form (BMV 2310) (https://publicsafety.ohio.gov/static/bmv2310.pdf) via email to BMV2310@dps.ohio.gov, fax Attn: Special Case Unit (614) 308-5211, or mail Ohio BMV Attn: Special Case Unit P.O. Box 16784 Columbus, OH 43216-6784. For questions, use Ohio BMV live chat services.
**Oklahoma**		Send a completed Request for Driver Review form (https://oklahoma.gov/content/dam/ok/en/dps/docs/requestfordriverreview-06.14.17.pdf) via mail to Attention: Medical Desk Department of Public Safety Driver Compliance Division - Medical Desk PO Box 11415 Oklahoma City, OK 73136-0415. For questions, call (405) 425-2083 or (405) 425-2059.
**Oregon**		For Mandatory Reporting, send in a completed Mandatory Impairment Referral Form (https://www.oregon.gov/odot/Forms/DMV/7230fill.pdf) via fax to (503) 945-5329 or mail to DMV - Driver Safety Unit 1905 Lana Avenue NE, Salem Oregon 97314. For voluntary reporting, send a completed Driver Evaluation Request Form (https://www.oregon.gov/odot/Forms/DMV/6066fill.pdf) via fax to (503) 945-5329 or mail to DMV Driver Specialty Services, 1905 Lana Avenue NE, Salem Oregon 97314 For questions, call (503) 945-5083 or the At-Risk Program Coordinator: (503) 945-5295.
**Pennsylvania**		Send in a completed Initial Reporting Form (DL-13) (https://www.dot.state.pa.us/public/dvspubsforms/BDL/BDL%20Form/DL-13.pdf) or one of the many medical condition-specific reports available on the DMV website (https://www.dmv.pa.gov/Information-Centers/Medical-Reporting/Pages/Medical-Reporting-Forms.aspx) or a written letter via fax to (717) 705-4415 or mail to PennDOT's Medical Unit at Bureau of Driver Licensing Driver Qualifications Section P.O. Box 68682 Harrisburg, Pennsylvania 17106-8682.
**Rhode Island**	X	Send a letter to the RI DMV via fax to (401) 462-0829 or mail to Attn: medical advisory board Rhode Island Division of Motor Vehicles Adjudication Office 600 New London Avenue, Cranston, RI 02920.

© 2024 Tran EM et al. *JAMA Network Open.*

**South Carolina**	X	Send a written letter on letterhead stating driver name and date of birth via fax to (803) 896-9926 or mail to Driver Improvement Office, PO box 1498 Blythewood, SC 29016.
**South Dakota**		Send a completed Medical Statement Form (https://dps.sd.gov/application/files/4415/0161/2419/Medical-Statement-2016.pdf) via fax to (605) 773-3018 or mail to South Dakota Department of Public Safety Driver Licensing Program 118 West Capitol Avenue Pierre, SD 57501. For questions, call the Driver Licensing Program at (605) 773-6883.
**Tennessee**		Send a written letter on letterhead via mail to the DMV 1150 Foster Avenue Nashville, TN 37243. For questions, call the Driver Improvement Section (615) 251-5235.
**Texas**		Send a completed form Examination/Investigation Request (DL-76) (https://www.dps.texas.gov/internetforms/getForm.ashx?id=DL-76.pdf) via email to MAB@DPS.Texas.gov, fax to (512) 424-5311, or mail to Texas Department of Public Safety, Enforcement and Compliance Service, P.O. Box 4087, Austin, TX 78773-0320.
**Utah**		Send a completed Functional Ability Evaluation Medical Report form (https://dld.utah.gov/wp-content/uploads/sites/17/2022/07/DLD-134-REV-4.22-.pdf).
**Vermont**	X	Send a written letter on letterhead via mail to 120 State St, Montpelier, VT 05603.
**Virginia**		Send a completed Medical Review Request Form (MED3) (https://www.dmv.virginia.gov/webdoc/pdf/med3.pdf) via fax to Medical Review Services (804) -367-1604 or mail to Department of Motor Vehicles Medical Review Services Post Office Box 27412 Richmond, Virginia 23269-0001. For questions, call (804) 367-6203
**Washington**		Send a completed Driver Evaluation Request Form (https://www.dol.wa.gov/forms/500008.pdf) via fax to (360) 570-7893 or mail to Restricted Licensing Department of Licensing PO Box 9030 Olympia, WA 98507. For questions, email MedicalCerts@dol.wa.gov.
**West Virginia**		Send in a completed Medical Review Request Form (https://transportation.wv.gov/DMV/DMVFormSearch/DMV-50-CD_medical_review_request_wf.pdf) or written letter on letterhead or a prescription pad via fax to (304) 957-0323 or mail to WV DMV Medical Review Unit PO BX 17030 Charleston, WV 25317. For questions, call (800) 642-9066.
**Wisconsin**		Send in page 2 of the Driver Condition or Behavior Report Form (MV314) (https://wisconsindot.gov/Documents/formdocs/mv3141.pdf) in addition to Pledge of Confidentiality Form (https://wisconsindot.gov/Pages/dmv/license-drvs/mdcl-cncrns/citizens.aspx) via email to dmvmedical@dot.wi.gov, fax to (608) 267-0518, or mail to Wisconsin Department of Transportation Medical Review P.O. Box 7918 Madison, WI 53707-7918. For questions, call (608) 266-2327.
**Wyoming**		Send a completed Driver Medical Evaluation Form (https://www.dot.state.wy.us/files/live/sites/wydot/files/shared/Driver_Services/Driver%20Medical%20Evaluation%20(FSDI-915)%20102016.pdf) via email to dot-medicals@wyo.gov, fax to (307) 777-4922, mail to Wyoming Department of Transportation (WYDOT) Driver Services - Driver Review Section 5300 Bishop Boulevard, Cheyenne, WY 82009-3340 OR Fax to: (307) 777-4922. For questions, call a Driver Review Representative at (307) 777-4839.
**No. (%) (n=50)**	17 (34)	

© 2024 Tran EM et al. *JAMA Network Open.*
