# Supplementary material for: Reporting Requirements, Confidentiality, and Legal Immunity for Physicians Who Report Medically Impaired Drivers
Source: JAMA Netw Open. 2024 Jan 5;7(1):e2350495. doi: 10.1001/jamanetworkopen.2023.50495 (PMC10770772; doi:10.1001/jamanetworkopen.2023.50495)
Supplement: Supplement 2. — Data Sharing Statement [file jamanetwopen-e2350495-s002.pdf]

Data Sharing Statement

Tran. Reporting Requirements, Confidentiality, and Legal Immunity for Physicians Who Report Medically Impaired Drivers. *JAMA Netw Open*. Published January 05, 2024. doi:10.1001/jamanetworkopen.2023.50495

Data
Data available: Yes
Data types: Data (not involving human participants)
How to access data: All data is presented in the manuscript tables.
When available: With publication

Supporting Documents
Document types: None

Additional Information
Who can access the data: Anyone requesting the data.
Types of analyses: For any purpose.
Mechanisms of data availability: All data is presented in the manuscript tables.
